# Prognostic Stratification in High‐Volume Metastatic Hormone‐Sensitive Prostate Cancer Treated With First‐Line Androgen Receptor Pathway Inhibitor–Based Combination Therapy

**DOI:** 10.1111/iju.70646

**Published:** 2026-09-21

**Authors:** Keiichiro Miyajima, Takafumi Yanagisawa, Wataru Fukuokaya, Ryoko Sakakibara, Naoki Uchida, Marcin Miszczyk, Shota Inoue, Akihiro Matsukawa, Fumihiko Urabe, Keiichiro Mori, Naoki Fujita, Shintaro Narita, Shingo Hatakeyama, Tomonori Habuchi, Shahrokh F. Shariat, Takahiro Kimura, JHA Collaborative Group

**Affiliations:** ^1^ Department of Urology, Comprehensive Cancer Center Medical University of Vienna Vienna Austria; ^2^ Department of Urology The Jikei University School of Medicine Tokyo Japan; ^3^ Collegium Medicum ‐ Faculty of Medicine WSB University Dąbrowa Górnicza Poland; ^4^ Department of Urology Okayama University Graduate School of Medicine, Dentistry and Pharmaceutical Sciences Okayama Japan; ^5^ Department of Urology Hirosaki University Graduate School of Medicine Hirosaki Japan; ^6^ Department of Urology Akita University School of Medicine Akita Japan; ^7^ Department of Urology Weill Cornell Medical College New York New York USA; ^8^ Department of Urology University of Texas Southwestern Medical Center Dallas Texas USA; ^9^ College of Medical Health Technologies Al‐Turath University Baghdad Iraq; ^10^ Hourani Center for Applied Scientific Research Al‐Ahliyya Amman University Amman Jordan; ^11^ Karl Landsteiner Institute of Urology and Andrology Vienna Austria; ^12^ Institute for Urology and Reproductive Health, I.M. Sechenov First Moscow State Medical University Moscow Russia

**Keywords:** androgen receptor pathway inhibitor, high‐volume disease, metastatic hormone‐sensitive prostate cancer, prognostic model, risk stratification

## Abstract

**Objectives:**

We aimed to stratify clinical risk among patients with high‐volume metastatic hormone‐sensitive prostate cancer (mHSPC) treated with first‐line androgen receptor pathway inhibitor (ARPI) plus androgen deprivation therapy (ADT).

**Methods:**

We retrospectively analyzed 626 patients with high‐volume mHSPC treated with ARPI+ADT between November 2016 and April 2025. A multivariable Cox model for time to castration‐resistant prostate cancer (TTCRPC) was developed using nine baseline characteristics, including age, hemoglobin, ECOG performance status, clinical T stage, ISUP grade group, EOD score, lung and liver metastases, and LDH, selected based on clinical relevance and prior evidence. Model discrimination and calibration were assessed using bootstrap internal validation. Patients were stratified into three risk groups based on the linear predictor, and associations with TTCRPC and overall survival (OS) were evaluated.

**Results:**

The median follow‐up was 34.8 months. Clinical T4 stage, ISUP grade group 5, and higher LDH were independently associated with shorter TTCRPC, whereas lung metastasis was associated with longer TTCRPC. The model had an optimism‐corrected C‐index of 0.69. TTCRPC differed significantly across the low‐, intermediate‐, and high‐risk groups (*p* < 0.001); compared with the low‐risk group, the HRs were 1.48 (95% CI 1.00–2.17) for the intermediate‐risk group and 4.09 (95% CI 2.90–5.77) for the high‐risk group. OS also differed significantly across the three risk groups (*p* < 0.001).

**Conclusions:**

In patients with high‐volume mHSPC treated with ARPI+ADT, the model showed modest discrimination for TTCRPC and identified groups with different TTCRPC and OS outcomes. These findings suggest the presence of clinically heterogeneous subgroups within high‐volume mHSPC and warrant independent validation in external cohorts.

## 1 Introduction

High‐volume metastatic hormone‐sensitive prostate cancer (mHSPC), as defined by the CHAARTED criteria [1], is a clinically important disease category that is currently used to guide treatment selection in major clinical guidelines [2, 3, 4]. For patients with high‐volume mHSPC, androgen receptor pathway inhibitor (ARPI) + androgen deprivation therapy (ADT) and docetaxel (DOC) + ARPI + ADT have become the standard of care. However, substantial heterogeneity in clinical outcomes persists even within this high‐volume population, underscoring the need for refined prognostic stratification beyond disease volume alone.

Among patients classified as high‐volume, visceral metastases have traditionally been regarded as a key adverse prognostic feature and represent one potential source of clinical heterogeneity within this population [5]. However, accumulating evidence suggests that the prognostic impact of visceral metastases is not uniform. In particular, liver metastases have consistently been associated with poor outcomes, whereas lung metastases may not independently confer an adverse prognosis [6, 7, 8, 9]. These observations highlight the broader limitations of relying solely on tumor volume or metastatic patterns to characterize clinical risk and suggest that additional risk stratification based on baseline clinical characteristics may provide further prognostic insight.

In this context, the present study aimed to determine whether baseline clinical characteristics can further stratify outcomes among patients with high‐volume mHSPC treated with contemporary first‐line systemic therapies. We developed a clinically grounded risk stratification model in patients treated with ARPI + ADT and exploratorily assessed its applicability in those receiving DOC + ARPI + ADT.

## 2 Materials and Methods

### 2.1 Patients and Treatment

This retrospective multicenter study evaluated a consecutive series of 1 262 patients with mHSPC who received first‐line systemic therapy between November 2016 and April 2025. Patients were treated at three academic centers—the Jikei University School of Medicine, Hirosaki University School of Medicine, and Akita University School of Medicine—and their affiliated institutions. First‐line systemic therapy consisted of ADT combined with ARPI, DOC, or both. ARPI + ADT included enzalutamide, apalutamide, or abiraterone in combination with ADT. DOC + ARPI + ADT consisted of DOC + darolutamide + ADT. Treatment dates and disease status at DOC initiation were reviewed to confirm that DOC was administered during the hormone‐sensitive phase.

**FIGURE 1:**
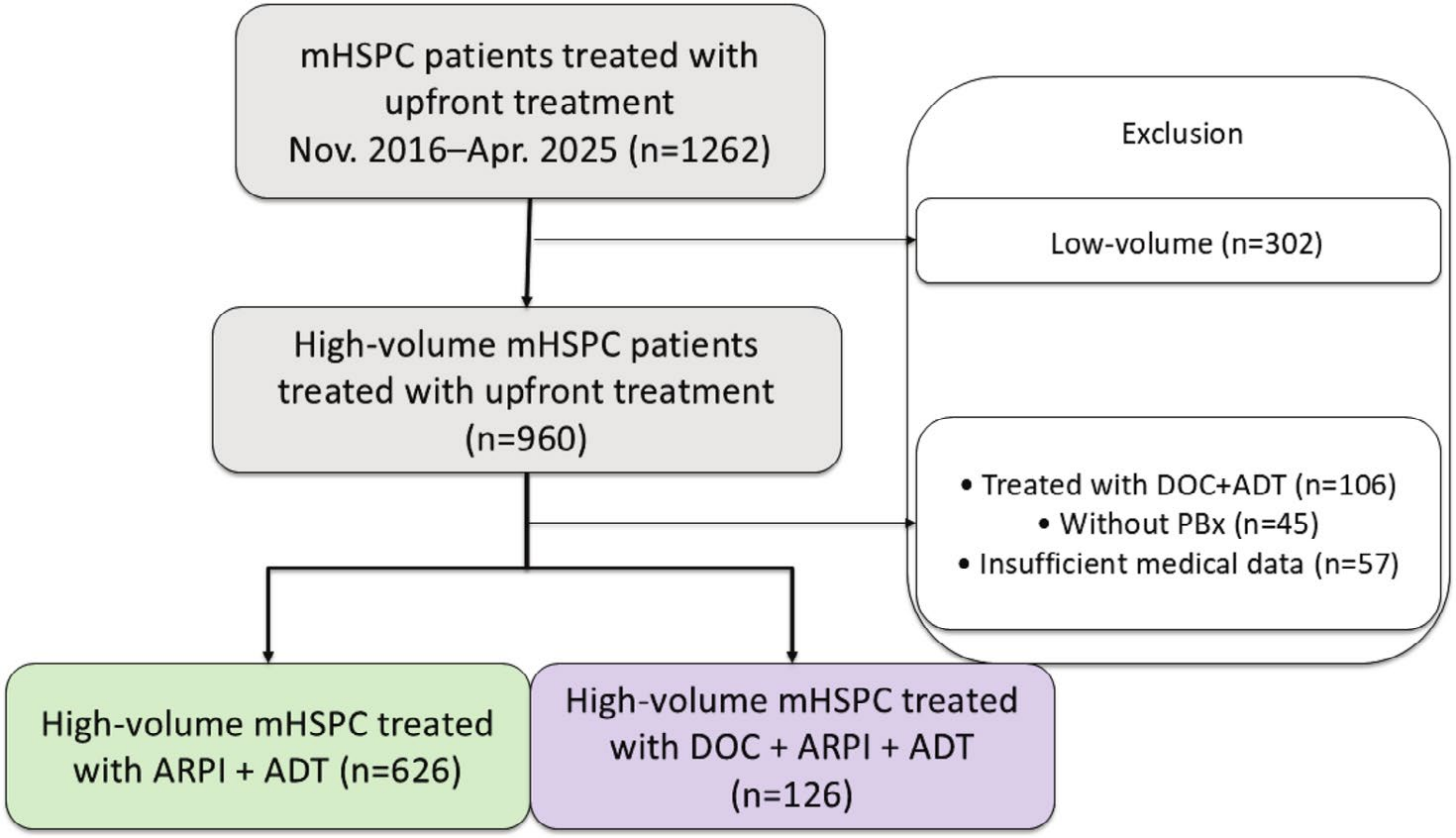
Study flow diagram of patient selection and treatment allocation. The diagram shows patient selection and treatment allocation among patients with high‐volume metastatic hormone‐sensitive prostate cancer (mHSPC) treated with first‐line systemic therapy. High‐volume disease was defined according to the CHAARTED criteria. Abbreviations: ARPI, androgen receptor pathway inhibitor; ADT, androgen deprivation therapy; DOC, docetaxel; PBx, prostate biopsy.

Disease volume was classified according to the CHAARTED criteria, and patients with low‐volume disease were excluded (*n* = 302). Among the remaining 960 patients with high‐volume mHSPC, we further excluded patients treated with DOC + ADT (*n* = 106), those who did not undergo prostate biopsy (*n* = 45), and those with insufficient baseline clinical data (*n* = 57), as DOC + ADT is not the preferred first‐line option for high‐volume mHSPC in current clinical guidelines [3, 4]. The final analytic cohort consisted of 752 patients with high‐volume mHSPC, including 626 patients treated with ARPI + ADT and 126 patients treated with DOC + ARPI + ADT (Figure 1). The study protocol was approved by the Institutional Review Board of Jikei University (no. 31–478[10060]).

### 2.2 Clinicopathological Data and Study Outcomes

For each patient, we retrospectively reviewed clinical records and extracted available clinical and baseline laboratory variables. These variables included age at diagnosis, baseline levels of hemoglobin (Hb), prostate‐specific antigen (PSA), lactate dehydrogenase (LDH), ECOG performance status (ECOG PS), clinical T stage, International Society of Urological Pathology Grade Group (ISUP GG), extent of disease (EOD) score, and presence of lung or liver metastases. ECOG PS and EOD score were assessed at the diagnosis of mHSPC before initiation of systemic therapy, whereas ISUP GG was based on the pathological assessment at the initial diagnosis of prostate cancer. EOD score was assessed based on bone scan to quantify the burden of bone metastases [10]. The first‐line treatment regimen (ARPI + ADT or DOC + ARPI + ADT) was also recorded.

The primary outcome of this study was time to castration‐resistant prostate cancer (TTCRPC), and the secondary endpoint was overall survival (OS). TTCRPC was measured from the start of ADT to the date of CRPC diagnosis. Patients who did not develop CRPC were censored at the date of last follow‐up or death, as applicable. In a complementary competing‐risk analysis, death before CRPC was treated as a competing event. OS was measured from ADT initiation to death from any cause. In principle, ADT was initiated before the addition of ARPI or DOC. Patients who were alive at the last follow‐up were censored at that date.

CRPC was diagnosed according to the European Association of Urology criteria, requiring castrate serum testosterone level < 50 ng/dL (< 1.7 nmol/L) together with biochemical, radiographic, or unequivocal clinical progression. Biochemical progression was defined as three consecutive rises in PSA at least 1 week apart, resulting in two 50% increases over the nadir with a PSA level > 2 ng/mL. Radiographic progression was defined as the appearance of at least two new bone lesions on bone scan or a new or progressive soft‐tissue lesion according to RECIST criteria [3, 11, 12].

Follow‐up imaging with CT and/or bone scan was generally performed every 6 to 12 months, with timing determined by symptoms, PSA kinetics, and physician judgment. PSA and routine laboratory tests were assessed at baseline and repeatedly during follow‐up according to routine clinical practice.

### 2.3 Statistical Analysis

Continuous variables were reported as medians with interquartile ranges (IQRs), and categorical variables as numbers with percentages. Baseline characteristics were compared between the two treatment cohorts using the Wilcoxon rank‐sum test for continuous variables and Fisher's exact test for categorical variables. For comparisons across the three individual ARPIs, the Kruskal–Wallis test was used for continuous variables and Fisher's exact test for categorical variables. Among patients who developed CRPC, the receipt of subsequent systemic therapy across risk groups was compared using Fisher's exact test; Monte Carlo simulation was used for the comparison of therapy types because of sparse cell counts. Median follow‐up duration and its 95% confidence interval (CI) were estimated using the reverse Kaplan–Meier method [13]. Reverse Kaplan–Meier curves with numbers at risk were generated according to treatment cohort. TTCRPC and OS were summarized using Kaplan–Meier estimates and compared with the log‐rank test. Survival estimates with corresponding 95% CIs were reported as median survival times or 2‐year survival rates, as appropriate.

A prognostic model for TTCRPC was constructed in the ARPI + ADT cohort using a multivariable Cox model. Candidate predictors were selected based on clinical relevance and prior evidence: Age, Hb level, ECOG PS, clinical T stage, ISUP GG, EOD score, lung metastasis, liver metastasis, and LDH [9, 14, 15, 16, 17, 18, 19]. Lung and liver metastases were evaluated separately because prior evidence suggests that these metastatic sites may have distinct prognostic implications in mHSPC. LDH was entered into the model as a natural log‐transformed continuous variable. Model performance was evaluated using the C‐index and internally validated with 1000 bootstrap resamples to estimate optimism‐corrected discrimination and calibration slope [20]. Variable selection based on statistical significance was not performed. For each patient, a linear predictor (LP) was calculated from the Cox model coefficients. Patients in the ARPI + ADT cohort were stratified into three prognostic groups according to tertiles of the LP: Low‐risk (LP≤first tertile), intermediate‐risk (first tertile<LP ≤ second tertile), and high‐risk (LP>second tertile). Pairwise comparisons between risk groups were additionally performed using Cox proportional hazards models. To assess the robustness of the primary TTCRPC analysis, a complementary competing‐risk analysis was performed with death before CRPC as a competing event. Cumulative incidence functions of CRPC were compared across risk groups using Gray's test, and subdistribution hazard ratios (sHRs) between risk groups were estimated using Fine–Gray regression.

As exploratory analyses, the same LP formula and cutoff values were applied to the DOC + ARPI + ADT cohort without model refitting or recalibration. This exploratory analysis was performed to assess whether the prognostic stratification developed in the ARPI + ADT cohort showed a similar pattern in patients treated with DOC + ARPI + ADT. Analyses were performed using R version 4.5.0 (R Foundation for Statistical Computing, Vienna, Austria). Statistical tests were two‐sided, and *p* < 0.05 was considered statistically significant.

## 3 Results

### 3.1 Patient Characteristics and the Overall Cohort Outcomes

Table 1 summarizes the baseline characteristics of the overall population, comprising 626 patients treated with ARPI + ADT and 126 patients treated with DOC + ARPI + ADT. Among patients in the ARPI + ADT cohort, 289 received abiraterone, 178 enzalutamide, and 159 apalutamide. Baseline characteristics according to the individual ARPI administered are presented in Table S1. All patients in the DOC + ARPI + ADT cohort initiated DOC within 3 months after ADT initiation, and none developed CRPC before DOC initiation. The median age of the overall cohort was 73 years (IQR 68–78). An EOD score of 4 was observed in 11% of patients, while lung and liver metastases were present in 25% and 3.1% of patients, respectively. Significant differences between the ARPI + ADT and DOC + ARPI + ADT cohorts were observed for age and the proportion of patients with EOD score 4 (*p* < 0.001 and *p* = 0.02, respectively). Subsequent systemic therapies administered after progression to CRPC are summarized in Table S2A.

**TABLE 1:** Baseline characteristics of the patients.

Characteristic	Overall *N* = 752^1^	ARPI + ADT *N* = 626^1^	DOC + ARPI + ADT *N* = 126^1^	p‐value^2^
Age	73 [68, 78]	73 [69, 79]	71 [66, 75]	< 0.001
Hb, g/dL	13.2 [11.8, 14.4]	13.2 [11.6, 14.4]	13.3 [12.1, 14.6]	0.1
PSA, ng/mL	341.7 [82.1, 1059.0]	346.5 [84.5, 1041.4]	307.0 [74.7, 1299.9]	0.8
LDH, U/L	202.0 [172.0, 256.0]	201.5 [173.0, 255.0]	206.5 [170.0, 278.0]	0.5
ECOG PS ≥ 2	62 (8.2)	53 (8.5)	9 (7.1)	0.7
Clinical T Stage 4	248 (33)	201 (32)	47 (37)	0.3
ISUP GG 5	472 (63)	401 (64)	71 (56)	0.1
EOD score 4	83 (11)	61 (9.7)	22 (17)	0.02
Lung metastasis	188 (25)	155 (25)	33 (26)	0.7
Liver metastasis	23 (3.1)	16 (2.6)	7 (5.6)	0.09

Abbreviations: ADT, androgen deprivation therapy; ARPI, androgen receptor pathway inhibitor; DOC, docetaxel; Hb, hemoglobin; PSA, prostate‐specific antigen; LDH, lactate dehydrogenase; ECOG PS, Eastern Cooperative Oncology Group performance status; ISUP GG, International Society of Urological Pathology grade group; EOD, extent of disease.

^1^ Median [Q1, Q3]; *n* (%).

^2^ Wilcoxon rank‐sum test; Fisher’s exact test.

Follow‐up duration and event summary are presented in Table S3. The median follow‐up duration was 34.8 months (95% CI 31.4–37.3) in the ARPI + ADT cohort, and 12.9 months (95% CI 11.0–15.7) in the DOC + ARPI + ADT cohort. Overall, 239 patients developed CRPC and 164 patients died during follow‐up. Figure S1 shows the reverse Kaplan–Meier curves for follow‐up duration according to treatment cohort.

### 3.2 Development and Performance of a Prognostic Model for TTCRPC

In multivariable Cox regression analysis, clinical T Stage 4, ISUP GG 5, and elevated LDH were identified as independent predictors of shorter TTCRPC, whereas lung metastasis was independently associated with longer TTCRPC (Table 2). The model demonstrated an apparent C‐index of 0.71 (95% CI 0.67–0.74) and an optimism‐corrected C‐index of 0.69 for TTCRPC after bootstrap validation. The optimism‐corrected calibration slope was 0.90. The complete LP formula, regression coefficients, variable coding, and risk‐group cutoff values are provided in Table S4.

**TABLE 2:** Multivariable Cox proportional hazards analysis of baseline factors for time to castration‐resistant prostate cancer in patients with high‐volume metastatic hormone‐sensitive prostate cancer treated with ARPI plus ADT.

Characteristic	HR	95% CI	p‐value
Age, per 1‐year increase	1.02	0.997–1.04	0.1
Hb, per 1‐g/dL increase	0.94	0.88–1.01	0.08
ECOG PS (≥ 2 vs. 0–1)	1.25	0.79–1.99	0.3
Clinical T Stage (4 vs. ≤ 3)	1.47	1.10–1.95	0.008
ISUP GG (5 vs. ≤ 4)	1.76	1.29–2.40	< 0.001
EOD score (4 vs. ≤ 3)	0.80	0.52–1.22	0.3
Lung metastasis (Yes vs. No)	0.71	0.51–0.99	0.04
Liver metastasis (Yes vs. No)	0.81	0.38–1.72	0.6
ln(LDH)	3.86	2.71–5.51	< 0.001

*Note:* The HRs for age and Hb represent the change in hazard per 1‐year and 1‐g/dL increase, respectively. LDH was entered into the model as a natural log‐transformed continuous variable.

Abbreviations: CI, confidence interval; EOD, extent of disease; ECOG PS, Eastern Cooperative Oncology Group performance status; Hb, hemoglobin; HR, hazard ratio; ISUP GG, International Society of Urological Pathology Grade Group; LDH, lactate dehydrogenase.

In a post hoc analysis, a simplified model comprising the four independently associated predictors retained most of the discrimination of the full model (optimism‐corrected C‐index, 0.68 vs. 0.69; Table S5).

### 3.3 Risk Stratification in the ARPI + ADT Cohort

**FIGURE 2:**
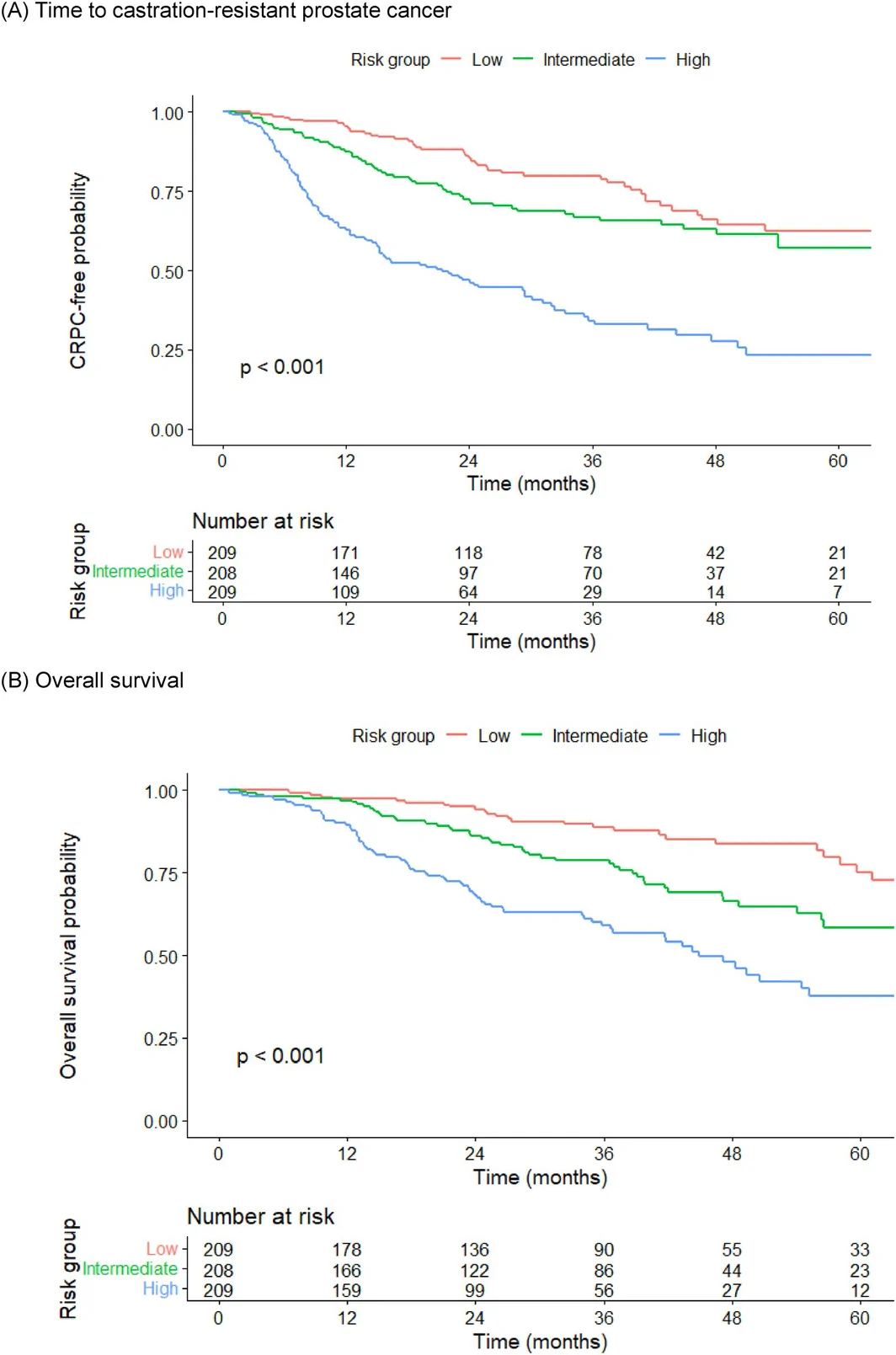
Kaplan–Meier curves for time to castration‐resistant prostate cancer and overall survival stratified by risk groups in the ARPI + ADT cohort. (A) Time to castration‐resistant prostate cancer and (B) overall survival. Patients were stratified into low‐, intermediate‐, and high‐risk groups based on the linear predictor derived from the optimism‐corrected prognostic model. Numbers at risk are shown below the x‐axis. *p* values were calculated using the log‐rank test.

Using the LP derived from the multivariable Cox model, patients were stratified into three risk groups. In the ARPI + ADT cohort, TTCRPC differed significantly across the three risk groups (*p* < 0.001; Figure 2A). In pairwise Cox regression analyses, the intermediate‐risk group had shorter TTCRPC than the low‐risk group (HR 1.48, 95% CI 1.00–2.17; *p* = 0.048). The high‐risk group had substantially shorter TTCRPC than both the low‐risk (HR 4.09, 95% CI 2.90–5.77; *p* < 0.001) and intermediate‐risk groups (HR 2.77, 95% CI 2.02–3.80; *p* < 0.001). The median TTCRPC was not reached in the low‐ and intermediate‐risk groups, whereas it was 21.4 months (95% CI 15.3–30.2) in the high‐risk group. The 2‐year CRPC‐free rates were 85% (95% CI 80%–91%), 73% (95% CI 66%–80%), and 47% (95% CI 40%–55%) in the low‐, intermediate‐, and high‐risk groups, respectively.

In a complementary competing‐risk analysis, 52 patients died before documented CRPC. The cumulative incidence of CRPC differed significantly across the three risk groups (*p* < 0.001; Figure S2). Compared with the low‐risk group, the high‐risk group had a significantly higher subdistribution hazard of CRPC (sHR 3.76, 95% CI 2.71–5.23; *p* < 0.001), whereas the difference between the intermediate‐ and low‐risk groups was not statistically significant (sHR 1.41, 95% CI 0.96–2.05; *p* = 0.08). The high‐risk group also had a significantly higher subdistribution hazard than the intermediate‐risk group (sHR 2.67, 95% CI 1.94–3.68; *p* < 0.001).

With respect to OS, survival differed significantly across the three risk groups (*p* < 0.001; Figure 2B). In pairwise Cox regression analyses, the intermediate‐risk group had significantly worse OS than the low‐risk group (HR 2.18, 95% CI 1.36–3.51; *p* = 0.001). The high‐risk group had significantly worse OS than both the low‐risk group (HR 4.35, 95% CI 2.79–6.80; *p* < 0.001) and the intermediate‐risk group (HR 1.99, 95% CI 1.40–2.84; *p* < 0.001). The median OS was not reached in the low‐risk group, whereas it was 67.0 months (95% CI 56.6 months to not reached) in the intermediate‐risk group and 44.8 months (95% CI 36.8 months to not reached) in the high‐risk group. The corresponding 2‐year OS rates were 94% (95% CI, 91%–98%), 86% (95% CI, 81%–92%), and 68% (95% CI, 61%–76%) in the low‐, intermediate‐, and high‐risk groups, respectively.

Among patients who developed CRPC in the ARPI + ADT cohort, the proportion receiving subsequent systemic therapy did not differ significantly across the low‐, intermediate‐, and high‐risk groups (74%, 81%, and 83%, respectively; *p* = 0.4). The distribution of subsequent systemic therapy types was also comparable across the three risk groups (*p* = 0.3; Table S2B).

### 3.4 Exploratory Risk Stratification in the DOC + ARPI + ADT Cohort

In the exploratory application of the same risk stratification to the DOC + ARPI + ADT cohort, no statistically significant differences were observed among the three risk groups for either TTCRPC (*p* = 0.2; Figure S3A) or OS (*p* = 0.98; Figure S3B).

## 4 Discussion

This study evaluated whether baseline clinical variables could refine prognostic assessment among patients with high‐volume mHSPC receiving first‐line ARPI + ADT. The derived model showed modest discrimination for TTCRPC and stratified patients into risk groups with significantly different TTCRPC and OS. These findings indicate that substantial prognostic heterogeneity remains even among patients already classified as having high‐volume disease.

Specifically, clinical T Stage 4, ISUP GG 5, and elevated LDH were independently associated with shorter TTCRPC, whereas lung metastasis was associated with longer TTCRPC. These findings are consistent with previous real‐world studies and a recent systematic review and meta‐analysis showing the prognostic relevance of these features in patients with mHSPC [7, 8, 9, 15, 17, 19, 21, 22, 23]. In contrast, although liver metastases have been reported as an adverse feature in previous studies [9, 24], they did not reach statistical significance in the present analysis, which may reflect the limited number of such cases.

Several prognostic models have previously been proposed for mHSPC. Fujiwara et al. developed a four‐factor model for predicting cancer‐specific survival in patients with de novo mHSPC receiving ADT with or without first‐generation antiandrogens and subsequently evaluated its prognostic utility for TTCRPC in the ARPI + ADT cohort (*n* = 81) [25]. More recently, Koguchi et al. developed a three‐factor risk model for castration resistance‐free survival in patients with bone‐metastatic mHSPC treated with ARPI + ADT [26]. However, these models were not specifically developed in patients with high‐volume disease. The present study therefore differs by focusing exclusively on patients with high‐volume mHSPC treated with ARPI + ADT and by evaluating whether baseline clinical factors can further stratify TTCRPC within this high‐volume population. Its potential incremental value lies in providing additional group‐level prognostic stratification within a population already classified as high‐volume, rather than replacing existing classifications or demonstrating improved individual‐level predictive accuracy.

The present model was designed as a clinically grounded prognostic framework incorporating baseline clinical factors supported by prior evidence, rather than being optimized through data‐driven variable selection. Its optimism‐corrected C‐index of 0.69 indicates modest discriminatory performance. The distinction between the low‐ and intermediate‐risk groups for TTCRPC was modest and was attenuated in the competing‐risk analysis, whereas the high‐risk group consistently showed poorer outcomes. Although a post hoc simplified model based on the four independently associated predictors retained most of the discriminatory performance of the full model, it was derived within the same cohort. Therefore, the clinical utility of both models remains unestablished, and independent external validation is required before clinical application.

In contrast, the prognostic stratification was less apparent in patients treated with DOC + ARPI + ADT. Younger age and the higher prevalence of EOD score 4 in the DOC + ARPI + ADT cohort may reflect treatment selection in clinical practice. Because prognostic analyses were conducted separately within each treatment cohort, these baseline differences are unlikely to affect within‐cohort risk stratification, although they may limit direct comparisons between the two cohorts. It remains uncertain whether the less apparent separation reflects greater treatment intensity, differences in patient characteristics, or simply limited statistical precision. Given the limited follow‐up and small number of events, these exploratory findings should be regarded as hypothesis‐generating.

This study has several limitations. First, the relatively short follow‐up duration and limited number of events, particularly in the DOC + ARPI + ADT cohort, limited the precision of outcome estimates. This limitation is especially relevant to OS, for which longer follow‐up and additional events are needed. Second, this was a retrospective, non‐randomized study, and therefore residual confounding and selection bias cannot be fully excluded. Surveillance imaging schedules and post‐CRPC therapeutic regimens were not standardized across institutions, which may have influenced TTCRPC and OS ascertainment, respectively. Third, although the prognostic model underwent internal validation using bootstrap resampling, the model lacks external validation. Fourth, the analysis was restricted to patients with high‐volume disease, which limits its generalizability to other populations. Fifth, the model did not include emerging molecular or imaging biomarkers, which may have constrained the predictive performance of the model. Finally, risk stratification was based on tertiles of the LP, which may not represent clinically optimal cut‐off values and could reduce reproducibility across different cohorts.

In patients with high‐volume mHSPC, a prognostic model based on established baseline clinical factors demonstrated modest discrimination for TTCRPC and stratified TTCRPC and OS in those treated with ARPI + ADT. These findings highlight prognostic heterogeneity within high‐volume mHSPC and support further investigation of risk stratification frameworks in this population.

## Author Contributions

**Takafumi Yanagisawa:** conceptualization, methodology, supervision. **Keiichiro Mori:** writing – review and editing. **Shintaro Narita:** writing – review and editing. **Ryoko Sakakibara:** writing – review and editing. **Wataru Fukuokaya:** methodology. **Keiichiro Miyajima:** methodology, formal analysis, investigation, writing – original draft. **Marcin Miszczyk:** writing – review and editing. **Shota Inoue:** writing – review and editing. **Naoki Uchida:** writing – review and editing. **Shingo Hatakeyama:** writing – review and editing. **Fumihiko Urabe:** writing – review and editing. **Akihiro Matsukawa:** writing – review and editing. **Shahrokh F. Shariat:** writing – review and editing. **Tomonori Habuchi:** writing – review and editing. **Takahiro Kimura:** writing – review and editing, supervision. **Naoki Fujita:** writing – review and editing.

## Funding

The authors have no reports.

## Ethics Statement

This study was approved by the Institutional Review Board of The Jikei University School of Medicine (approval no. 31–478 [10060]).

## Consent

Patient consent was waived due to the retrospective nature of the study and the use of anonymized data.

## Conflicts of Interest

Author Shahrokh F. Shariat received the following honoraria: Astellas, AstraZeneca, BMS, Ferring, Ipsen, Janssen, MSD, Olympus, Pfizer, Roche, and Takeda. Consulting or advisory role: Astellas, AstraZeneca, BMS, Ferring, Ipsen, Janssen, MSD, Olympus, Pfizer, Pierre Fabre, Roche, and Takeda.

Speakers' bureau: Astellas, AstraZeneca, Bayer, BMS, Ferring, Ipsen, Janssen, MSD, Olympus, Pfizer, Richard Wolf, Roche, and Takeda. Takahiro Kimura is a paid consultant/advisor of Astellas, AstraZeneca, Bayer, Janssen, Sanofi, and Takeda. Shintaro Narita received honoraria from Janssen Pharmaceutical K.K., Takeda Pharmaceutical Company Ltd., Astellas Pharma Inc., AstraZeneca K.K., Kyorin, Sysmex, and Bayer AG. Other authors declare they have no financial interests.

## Supporting information


**Table S1:** Baseline characteristics according to the individual ARPI administered in the ARPI + ADT cohort.
**Table S2:** Subsequent systemic therapies after progression to castration‐resistant prostate cancer according to treatment cohort and risk group.
**Table S3:** Follow‐up duration and event summary by treatment cohort.
**Table S4:** Regression coefficients and coding of variables included in the model.
**Table S5:** Comparison of predictive performance between the full and simplified prognostic models.
**Figure S1:** Reverse Kaplan–Meier curves for follow‐up duration according to treatment cohort.
**Figure S2:** Cumulative incidence of castration‐resistant prostate cancer according to risk group, with death before CRPC treated as a competing event.
**Figure S3:** Kaplan–Meier curves for time to castration‐resistant prostate cancer and overall survival stratified by risk groups in the DOC + ARPI + ADT cohort.

## Data Availability

The data that support the findings of this study are available on request from the corresponding author. The data are not publicly available due to privacy or ethical restrictions.
