# Supplementary material for: Prognostic Stratification in High‐Volume Metastatic Hormone‐Sensitive Prostate Cancer Treated With First‐Line Androgen Receptor Pathway Inhibitor–Based Combination Therapy
Source: Int J Urol. 2026 Sep 21;33(9):e70646. doi: 10.1111/iju.70646 (PMC13591654; doi:10.1111/iju.70646)
Supplement: Supplementary file 1 [file IJU-33-0-s001.docx]

**Supplementary Information**

**Supplementary Table 1.** Baseline characteristics according to the individual ARPI administered in the ARPI + ADT cohort

**Supplementary Table 2.** Subsequent systemic therapies after progression to castration-resistant prostate cancer according to treatment cohort and risk group

**Supplementary Table 3.** Follow-up duration and event summary by treatment cohort

**Supplementary Table 4.** Regression coefficients and coding of variables included in the model

**Supplementary Table 5**. Comparison of predictive performance between the full and simplified prognostic models

**Supplementary Figure 1.** Reverse Kaplan–Meier curves for follow-up duration according to treatment cohort

**Supplementary Figure 2.** Cumulative incidence of castration-resistant prostate cancer according to risk group, with death before CRPC treated as a competing event

**Supplementary Figure 3.** Kaplan–Meier curves for time to castration-resistant prostate cancer and overall survival stratified by risk groups in the DOC + ARPI + ADT cohort

**Supplementary Table 1.** Baseline characteristics according to the individual ARPI administered in the ARPI + ADT cohort

| Characteristic | ARPI + ADT N = 626^1^ | Abiraterone N = 289^1^ | Enzalutamide N = 178^1^ | Apalutamide N = 159^1^ | p-value^2^ |
| --- | --- | --- | --- | --- | --- |
| Age | 73 [69, 79] | 72 [68, 77] | 75 [71, 80] | 73 [69, 79] | <0.001 |
| Hb, g/dL | 13.2 [11.6, 14.4] | 13.4 [12.3, 14.6] | 12.7 [11.0, 14.1] | 13.1 [11.5, 14.2] | 0.002 |
| PSA, ng/mL | 346.5 [84.5, 1041.4] | 377.0 [98.8, 1242.0] | 284.2 [60.0, 1051.2] | 346.0 [95.3, 835.0] | 0.4 |
| LDH, U/L | 201.5 [173.0, 255.0] | 201.0 [173.0, 248.0] | 202.5 [169.0, 267.0] | 201.0 [173.0, 256.0] | >0.9 |
| ECOG PS ≥2 | 53 (8.5) | 17 (5.9) | 23 (13) | 13 (8.2) | 0.03 |
| Clinical T stage 4 | 201 (32) | 99 (34) | 48 (27) | 54 (34) | 0.2 |
| ISUP GG 5 | 401 (64) | 182 (63) | 113 (63) | 106 (67) | 0.7 |
| EOD score 4 | 61 (9.7) | 32 (11) | 14 (7.9) | 15 (9.4) | 0.5 |
| Lung metastasis | 155 (25) | 76 (26) | 47 (26) | 32 (20) | 0.3 |
| Liver metastasis | 16 (2.6) | 7 (2.4) | 6 (3.4) | 3 (1.9) | 0.7 |
| 1 : Median [Q1, Q3]; n (%), 2 : Kruskal-Wallis rank sum test; Fisher’s exact test Abbreviations: ADT, androgen deprivation therapy; ARPI, androgen receptor pathway inhibitor;Hb, hemoglobin; PSA, prostate-specific antigen; LDH, lactate dehydrogenase; ECOG PS, Eastern Cooperative Oncology Group performance status; ISUP GG, International Society of Urological Pathology grade group; EOD, extent of disease. | | | | | |

**Supplementary Table 2.** Subsequent systemic therapies after progression to castration-resistant prostate cancer according to treatment cohort and risk group

(A) Subsequent systemic therapies according to treatment cohort

|  | Overall  N = 752 | ARPI + ADT  N = 626 | DOC + ARPI + ADT  N = 126 |
| --- | --- | --- | --- |
| Progression to CRPC, n (%) | 239 (32) | 221 (35) | 18 (14) |
| Patients receiving subsequent systemic therapy, n (%)^1^ | 191 (80) | 178 (80) | 13 (72) |
| Subsequent systemic therapy |  |  |  |
| Docetaxel | 75 | 74 | 1 |
| Cabazitaxel | 4 | 1 | 3 |
| Enzalutamide | 59 | 55 | 4 |
| Abiraterone | 27 | 24 | 3 |
| Apalutamide | 2 | 2 | 0 |
| Ra-223 | 2 | 1 | 1 |
| Ra-223 + enzalutamide | 5 | 4 | 1 |
| Ra-223 + estramustine | 1 | 1 | 0 |
| Other hormonal therapy (including NSAA) | 7 | 7 | 0 |
| Other chemotherapy | 5 | 5 | 0 |
| Investigational drugs | 4 | 4 | 0 |
| ¹ Percentages are calculated among patients who developed CRPC.  Abbreviations: ADT, androgen deprivation therapy; ARPI, androgen receptor pathway inhibitor; CRPC, castration-resistant prostate cancer; DOC, docetaxel; NSAA, nonsteroidal antiandrogen; Ra-223, radium-223 dichloride. | | | |

(B) Subsequent systemic therapies according to risk group in the ARPI + ADT cohort

|  | ARPI + ADT N = 626 | Low risk N = 209 | Intermediate risk N = 208 | High risk N = 209 | p-value |
| --- | --- | --- | --- | --- | --- |
| Progression to CRPC, n (%) | 221 (35) | 46 (22) | 59 (28) | 116 (56) |  |
| Patients receiving subsequent systemic therapy, n (%)^1^ | 178 (81) | 34 (74) | 48 (81) | 96 (83) | 0.4 |
| Subsequent systemic therapy |  |  |  |  | 0.3 |
| Docetaxel | 74 | 12 | 22 | 40 |  |
| Cabazitaxel | 1 | 1 | 0 | 0 |  |
| Enzalutamide | 55 | 15 | 12 | 28 |  |
| Abiraterone | 24 | 2 | 7 | 15 |  |
| Apalutamide | 2 | 0 | 1 | 1 |  |
| Ra-223 | 1 | 0 | 0 | 1 |  |
| Ra-223 + enzalutamide | 4 | 0 | 0 | 4 |  |
| Ra-223 + estramustine | 1 | 1 | 0 | 0 |  |
| Other hormonal therapy (including NSAA) | 7 | 0 | 4 | 3 |  |
| Other chemotherapy | 5 | 2 | 1 | 2 |  |
| Investigational drugs | 4 | 1 | 1 | 2 |  |
| ¹ Percentages are calculated among patients who developed CRPC in each risk group. Differences in the receipt of subsequent systemic therapy across risk groups were evaluated using Fisher’s exact test. The distribution of subsequent therapy types was compared using Fisher’s exact test with Monte Carlo simulation because of sparse cell counts. Abbreviations: ADT, androgen deprivation therapy; ARPI, androgen receptor pathway inhibitor; CRPC, castration-resistant prostate cancer; NSAA, nonsteroidal antiandrogen; Ra-223, radium-223 dichloride. | | | | | |

**Supplementary Table 3.** Follow-up duration and event summary by treatment cohort

| Characteristic | Overall  N = 752 | ARPI + ADT  N = 626 | DOC + ARPI + ADT  N = 126 | p-value^1^ |
| --- | --- | --- | --- | --- |
| Median follow-up duration, months (95% CI) | 28.4 [26.4, 31.0] | 34.8 [31.4, 37.3] | 12.8 [11.0, 15.7] | <0.001 |
| CRPC events, n (%) | 239 (32) | 221 (35) | 18 (14) |  |
| Deaths, n (%) | 164 (22) | 156 (25) | 8 (6.3) |  |
| 1: Median follow-up duration was estimated using the reverse Kaplan–Meier method, and follow-up distributions between treatment cohorts were compared using the log-rank test. Abbreviations: ADT, androgen deprivation therapy; ARPI, androgen receptor pathway inhibitor; CI, confidence interval; CRPC, castration-resistant prostate cancer; DOC, docetaxel. | | | | |

**Supplementary Table 4.** Regression coefficients and coding of variables included in the model

| Predictor | Coding/unit | Reference | β coefficient |
| --- | --- | --- | --- |
| Age | per 1 year | — | 0.016064 |
| ECOG PS ≥2 | 1 = ≥2, 0 = 0–1 | 0–1 | 0.225966 |
| Hemoglobin | per 1 g/dL | — | -0.059554 |
| ISUP GG 5 | 1 = GG5, 0 = ≤GG4 | ≤GG4 | 0.565966 |
| Clinical T stage 4 | 1 = cT4, 0 = cT≤3 | cT≤3 | 0.383216 |
| EOD score 4 | 1 = EOD4, 0 = EOD≤3 | EOD≤3 | -0.228810 |
| Liver metastasis | 1 = Yes, 0 = No | No | -0.207651 |
| Lung metastasis | 1 = Yes, 0 = No | No | -0.342262 |
| ln(LDH) | natural logarithm of LDH (U/L) | — | 1.351890 |
| LP formula | | | |
| LP = 0.016064 × age + 0.225966 × I(ECOG PS ≥2) − 0.059554 × Hb + 0.565966 × I(ISUP GG 5) + 0.383216 × I(cT4) − 0.228810 × I(EOD 4) − 0.207651 × I(liver metastasis) − 0.342262 × I(lung metastasis) + 1.351890 × ln(LDH), where binary indicators were coded as 1 for the specified category and 0 for the corresponding reference category. | | | |
| Risk-group cutoffs | | | |
| Low risk | LP ≤ 7.792096 |  |  |
| Intermediate risk | 7.792096 < LP ≤ 8.287295 |  |  |
| High risk | LP > 8.287295 |  |  |
| For the DOC + ARPI + ADT cohort, the same LP formula, regression coefficients, and cutoff values derived from the ARPI + ADT cohort were applied without model refitting or recalibration.  Abbreviations: LP, linear predictor; ECOG PS, Eastern Cooperative Oncology Group performance status; Hb, hemoglobin; ISUP GG, International Society of Urological Pathology Grade Group; EOD, extent of disease; DOC, docetaxel; ARPI, androgen receptor pathway inhibitor; ADT, androgen deprivation therapy. | | | |

**Supplementary Table 5.** Comparison of predictive performance between the full and simplified prognostic models

| Model | Apparent C-index (95% CI) | Optimism-corrected C-index | Optimism-corrected calibration slope |
| --- | --- | --- | --- |
| Full 9-variable model | 0.71 (0.67-0.74) | 0.69 | 0.90 |
| Post hoc 4-variable model | 0.69 (0.65-0.72) | 0.68 | 0.96 |

**Supplementary Figure 1.** Reverse Kaplan–Meier curves for follow-up duration according to treatment cohort

**Supplementary Figure 2.** Cumulative incidence of castration-resistant prostate cancer according to risk group, with death before CRPC treated as a competing event

**Supplementary Figure 3.** Kaplan–Meier curves for time to castration-resistant prostate cancer and overall survival stratified by risk groups in the DOC + ARPI + ADT cohort

(A) Time to castration-resistant prostate cancer

(B) Overall survival
